# A Universal Formulary

**Published:** 1850-07

**Authors:** 


### ARTICLE III.

*A Universal Formulary: containing the methods of preparing and administering officinal and other medicines. The whole adapted to Physicians and Pharmaceutists:* By R. EGLESFIELD GRIFFITH, M. D.; pp. 567 ; Philadelphia, Lea & Blanchard, 1850. (From the Publishers, through J. Keen, Jr., & Bro., Chicago.

This seems to be a very comprehensive work, so far as the range of its articles and combinations is concerned, with a commendable degree of brevity and condensation in their explanation.

It cannot fail to be a useful and convenient book of reference to the two classes of persons to whom it particularly commends itself in the title page.

